# Oral fluoropyrimidines among the new drugs for patients with metastatic breast cancer

**DOI:** 10.1054/bjoc.2001.1819

**Published:** 2001-06

**Authors:** R C F Leonard

**Affiliations:** Department of Clinical Oncology, Western General Hospital, Edinburgh, UK

**Keywords:** breast cancer, anthracyclines, capecitabine, 5-FU, taxoids, vinorelbine, oral

## Abstract

Although drugs such as the taxoids and vinorelbine have increased the options available for anthracycline-resistant metastatic breast cancer, new therapeutic options are needed, particularly for taxoid-refractory tumours. Increasing emphasis is being placed on the development of oral agents, which many patients prefer provided efficacy is not compromised, particularly if the oral agents are less toxic than current intravenous agents. Capecitabine, a new, oral fluoropyrimidine, mimics continuous infusion 5-FU and is activated preferentially at the tumour site. Phase II studies of capecitabine have demonstrated encouraging response rates in patients with few further treatment options (20% response with an additional 43% achieving stable disease in paclitaxel-refractory patients; 36% response with a further 23% achieving stable disease in anthracycline-refractory patients). In addition, a randomized, phase II trial demonstrated a response rate of 30% (95% Cl: 19–43%) with capecitabine as first-line treatment for metastatic breast cancer, compared with 16% (95% Cl: 5–33%) in patients receiving low-dose CMF. These trials also showed that capecitabine has a favourable safety profile typical of infused fluoropyrimidines. Both alopecia and myelosuppression were rare. Capecitabine may therefore provide an effective, well-tolerated and convenient alternative to intravenous cytotoxic agents, not only in taxoid-resistant patients, but also in anthracycline-resistant metastatic breast cancer or as first-line therapy. Furthermore, the low incidence of myelosuppression makes capecitabine an attractive agent for incorporation into combination regimens with agents such as epirubicin/doxorubicin, the taxoids and vinorelbine. © 2001 Cancer ResearchCampaign http://www.bjcancer.com


*British Journal of Cancer* (2001) **84**(11), 1437–1442
© 2001 Cancer Research Campaign
doi: 10.1054/ bjoc.2001.1819 available online at http://www.idealibrary.com on IDEAL®

### Oral fluoropyrimidines among the new drugs for patients with metastatic breast cancer

**RCF Leonard**

Department of Clinical Oncology, Western General Hospital, Edinburgh, UK

**Summary** Although drugs such as the taxoids and vinorelbine have increased the options available for anthracycline-resistant metastatic breast cancer, new therapeutic options are needed, particularly for taxoid-refractory tumours. Increasing emphasis is being placed on the development of oral agents, which many patients prefer provided efficacy is not compromised, particularly if the oral agents are less toxic than current intravenous agents. Capecitabine, a new, oral fluoropyrimidine, mimics continuous infusion 5-FU and is activated preferentially at the tumour site. Phase II studies of capecitabine have demonstrated encouraging response rates in patients with few further treatment options (20% response with an additional 43% achieving stable disease in paclitaxel-refractory patients; 36% response with a further 23% achieving stable disease in anthracycline-refractory patients). In addition, a randomized, phase II trial demonstrated a response rate of 30% (95% CI: 19–43%) with capecitabine as first-line treatment for metastatic breast cancer, compared with 16% (95% CI: 5–33%) in patients receiving low-dose CMF. These trials also showed that capecitabine has a favourable safety profile typical of infused fluoropyrimidines. Both alopecia and myelosuppression were rare. Capecitabine may therefore provide an effective, well-tolerated and convenient alternative to intravenous cytotoxic agents, not only in taxoid-resistant patients, but also in anthracycline-resistant metastatic breast cancer or as first-line therapy. Furthermore, the low incidence of myelosuppression makes capecitabine an attractive agent for incorporation into combination regimens with agents such as epirubicin/doxorubicin, the taxoids and vinorelbine. © 2001 Cancer Research Campaign http://www.bjcancer.com

**Keywords:** breast cancer; anthracyclines; capecitabine; 5-FU; taxoids; vinorelbine; oral

Despite early diagnosis, a large proportion (up to 40%) of breast cancer patients will develop metastatic disease that is incurable with conventional treatments (Hortobagyi and Piccart-Gebhart, 1996). The choice of appropriate therapy for metastatic breast cancer is determined by several factors: pace of relapse (influenced in part by the interval between initial presentation and first relapse), hormone receptor status, previous treatment, presence or absence of visceral involvement, age and fitness (including co-morbid disease), patient preference and healthcare budgets. Conventional chemotherapy regimens based on cyclophosphamide, 5-fluorouracil (5-FU) and methotrexate (CMF) or an anthracycline (FAC, FEC) achieve response rates of 40–80% in chemotherapy-naïve patients, although further relapse is the rule, usually within months of stopping treatment. Moreover, the increasing use of chemotherapy, particularly anthracycline-based regimens, in the adjuvant setting means that new treatment options are required for metastatic disease. Several agents have been developed in recent years, including the taxoids (paclitaxel and docetaxel) and vinorelbine, and these have become the second-line treatments of choice in many countries (Livingston et al, 1997; Archer et al, 1998; Carlson, 1998; Ray-Coquard et al, 1998; Valero et al, 1998).

The oral fluoropyrimidine, capecitabine, has been developed as an enzymatically activated, tumour-selective agent, thereby increasing tumour concentrations of active drug and limiting exposure of normal tissue to the toxic effects of chemotherapy. Another important objective in developing capecitabine was to mimic continuous infusion 5-FU, for which response rates of 12% have been reported in heavily pretreated patients (Ng et al, 1994; Ragaz et al, 1997). This review examines oral fluoropyrimidines as an alternative or an addition to vinorelbine/taxoid therapy that offers metastatic breast cancer patients acceptable efficacy with good tolerability and ease of use. In particular, the role of capecitabine is examined as it offers the advantages of oral administration at home.

*Received 21 August 2000*
*Revised 30 January 2001*
*Accepted 20 February 2001*

*Correspondence to:* RCF Leonard

### NEW TREATMENT APPROACHES FOR METASTATIC BREAST CANCER

### Taxoids

The taxoids are being used increasingly, either alone or in combination with other agents, in breast cancer patients whose disease has relapsed after adjuvant chemotherapy or as first-line chemotherapy for metastatic disease. Studies in patients who have received extensive previous chemotherapy have shown response rates in the range of 20–38% with paclitaxel doses of 135–300 mg/m$^2$ every 3 weeks, and in the range of 35–58% with docetaxel doses of 100 mg/m$^2$ every 3 weeks (D'Andrea and Seidman, 1997). Two prospective, randomized, phase III trials have demonstrated that docetaxel achieves superior response rates compared with either mitomycin plus vinorelbine (Nabholtz et al, 1999) or doxorubicin monotherapy (Chan et al, 1999) in anthracycline-resistant breast cancer patients. The median time to disease progression and overall survival achieved with docetaxel were significantly longer than those achieved with mitomycin plus vinorelbine (Nabholtz et al, 1999) and equivalent to those achieved with doxorubicin (Chan et al, 1999).

A large number of studies have investigated paclitaxel in patients who have received little or no previous chemotherapy. Not surprisingly, response rates are higher than those seen when paclitaxel is used as second-line therapy (Seidman et al, 1995). Objective response rates range from 29–62%, and similar response rates have been reported to those of docetaxel (D'Andrea and Seidman, 1997). These response rates are similar to those achieved with conventional combination regimens used in breast cancer, although paclitaxel may be associated with less myelosuppression at equally cytotoxic doses (Bishop et al, 1997).

### Vinorelbine

To data there have been limited alternatives to paclitaxel and docetaxel in patients with relapsed or advanced breast cancer. Vinorelbine, a third-generation vinca alkaloid, has demonstrated good activity in advanced breast cancer, resulting in objective response rates of 16–17% in patients with anthracycline-resistant or -refractory disease (Degardin et al, 1994; Jones et al, 1995; Livingston et al, 1997), suggesting incomplete cross-resistance between vinorelbine and the anthracyclines. Objective response rates of up to 25% have been reported when vinorelbine is administered with granulocyte colony-stimulating factor support in relapsed patients (Livingston et al, 1997). As first- and second-line therapy, vinorelbine achieves objective response rates of 35– 41% (Fumoleau et al, 1993; Romero et al, 1994; Weber et al, 1995) and 24–32% (Roché et al, 1990; Weber et al, 1995), respectively. The dose-limiting toxicity in these studies was myelosuppression and other toxicities included phlebitis, peripheral neuropathy and myalgia. Many clinicians find that vinorelbine is an attractive alternative to taxoids in older or less fit patients.

It has been suggested from preclinical studies that a combination of taxoids and vinca alkaloids may be synergistic (Schiff and Horwitz, 1980) as a result of the differing activities of both types of agent on microtubule assembly. Studies of vinorelbine in combination with taxoids are ongoing (Livingston et al, 1997).

### 5-FU

The feasibility of treating anthracycline-refractory breast cancer patients with continuous 5-FU infusion has been demonstrated. Hansen et al (1987) reported that in 25 patients with extensive pretreatment (24 had received previous doxorubicin and 23 previous bolus 5-FU), who were treated with continuous low-dose 5-FU infusion, an objective response was achieved in 8 patients (32%). Side effects included hand-foot syndrome, mucositis, diarrhoea, and nausea and vomiting, but toxicity was easily manageable. The risk of these adverse effects is increased in patients with decreased dihydropyrimidine dehydrogenase (DPD) activity (Lu et al, 1998; Johnson et al, 1999). The efficacy of continuous 5-FU infusion reported in the study by Hansen et al was confirmed by Jabboury et al (1989) and Huan et al (1989). Unfortunately, continuous infusion therapy is labour intensive, inconvenient for the patient and is associated with complications and additional costs related to the need for central venous access. Therefore there is a strong medical need for an oral fluoropyrimidine which is capable of mimicking continuous 5-FU infusion.

Simple, convenient, oral chemotherapy regimens may be advantageous in many ways (DeMario and Ratain, 1998). In particular, a recent questionnaire-based study reported that most patients (89%) preferred oral to intravenous palliative chemotherapy provided that efficacy was maintained (Liu et al, 1997). Of the patients questioned in the study, 46% had previously received i.v. chemotherapy and 9% had previously been treated with oral agents, with 6% of patients having prior experience of both oral and i.v. chemotherapy. The main reasons for preferring oral chemotherapy were convenience, problems with intravenous lines and the ability to receive oral chemotherapy outside the clinic environment, indicating that patients with a limited life expectancy want to maintain as normal a life as possible.

### CAPECITABINE IN METASTATIC BREAST CANCER

The limitations of 5-FU prompted the development of a fluoropyrimidine derivative that could be given orally and would be activated preferentially in tumour tissue, thereby increasing tumour concentrations of active drug while minimizing exposure of normal tissue. Capecitabine is the first of a new class of fluoropyrimidines rationally designed as an oral, enzymatically activated fluoropyrimidine carbamate capable of mimicking continuous infusion 5-FU. The activation of capecitabine preferentially in tumour tissue results in higher intratumoral concentrations of 5-FU (Schüller et al, 2000).

Capecitabine is not cytotoxic itself and requires conversion to 5-FU through 3 sequential enzyme steps (Figure 1). The intermediates 5′-deoxy-5-fluorocytidine (5′-DFCR) and 5′-deoxy-5-fluorouridine (5'-DFUR) are also inactive. Thymidine phosphorylase, the enzyme involved in the final activation step, is active predominantly in tumour tissues, including colorectal, breast, cervical and ovarian cancers, and is present at significantly lower concentrations in adjacent normal tissue (Miwa et al, 1998). The carbamate structure of capecitabine allows the drug to be administered orally: absorption is rapid and extensive, and the compound passes unchanged into the liver for the first step in its activation (Budman et al, 1998; Judson et al, 1999).

Following administration of capecitabine to animals bearing human cancer xenografts, significantly higher concentrations of 5-FU were observed in tumour tissue (127-fold) than in plasma, but no selective distribution was seen following 5-FU administration (Ishikawa et al, 1998a). Capecitabine has shown significantly greater activity than 5-FU or UFT (uracil plus tegafur) in human tumour xenograft models when both drugs were administered at their maximum tolerated doses: tumour growth was inhibited by > 50% in 18 of 24 (75%) xenograft lines treated with capecitabine but in only one of 24 (4%) xenograft lines treated with 5-FU and 5

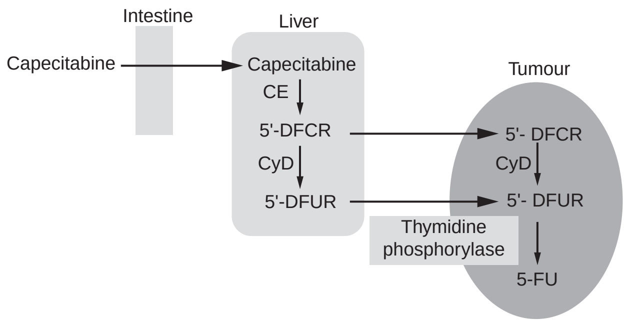

**Figure 1** Enzymatic activation of capecitabine to 5-FU via its inactive intermediates 5′-deoxy-5-fluorocytidine (5′-DFCR) and (5′-deoxy-5-fluorouridine (5′-DFUR)

 *© 2001 Cancer Research Campaign*

of 24 (21%) xenograft lines treated with UFT (Ishikawa et al, 1998b).

### Dose-finding studies of capecitabine as a single agent and in combination regimens

Capecitabine has been evaluated in a number of phase I clinical studies as either intermittent (2 weeks' treatment followed by 1 week's rest) (Mackean et al, 1998) or continuous monotherapy (Budman et al, 1998). It has also been studied in combination with leucovorin (Cassidy et al, 1998), paclitaxel (Khoury et al, 1998; Villalona-Calero et al, 1999) or docetaxel (Pronk et al, 2000) in patients with a range of locally advanced or metastatic tumours (predominantly breast and colorectal cancer) whose disease had relapsed or was resistant to conventional chemotherapy. As monotherapy, the maximum tolerated dose was found to be 3000 mg/m$^2$ daily for the intermittent regimen and 1657 mg/m$^2$ daily for the continuous regimen. The most common dose-limiting toxicities were diarrhoea, hand-foot syndrome, nausea and vomiting, and there was only minimal myelosuppression and no neurotoxicity at these doses. All toxicities were reversible. The doses recommended for phase II studies were 1255 mg/m$^2$ twice daily for the intermittent regimen and 667 mg/m$^2$ twice daily for the continuous regimen. The intermittent regimen was highly active and well tolerated, and allowed greater cumulative doses to be given as shown in a randomized, phase II trial (Van Cutsem et al, 2000).

As combination therapy with paclitaxel 175 mg/m$^2$ every 3 weeks, intermittent doses of capecitabine 1000 mg/m$^2$ twice daily were dose limiting in 3 of 6 patients treated. No dose-limiting toxicities were seen in 8 patients treated with capecitabine 825 mg/m$^2$ twice daily (Khoury et al, 1998). Response rates were promising in this study, with a partial response observed in 5 of the 10 evaluable patients.

A further phase I trial evaluated escalating doses of docetaxel (75, 85 and 100 mg/m$^2$ every 3 weeks) in combination with intermittent, fixed-dose capecitabine therapy (825 mg/m$^2$ twice daily for 2 weeks) in the first phase of the trial, followed by a second phase during which increased doses of capecitabine (1000 and 1250 mg/m$^2$ twice daily for 2 weeks) were given in combination with a fixed dose of docetaxel (Pronk et al, 2000). Grade 2 asthenia was considered to be dose limiting at the 1000/100 mg/m$^2$ dose level. The schedule of capecitabine 1250 mg/m$^2$ twice daily for 14 days plus docetaxel 75 mg/m$^2$ every 3 weeks was selected for further evaluation in a phase III trial, which has recently been reported (O'Shaughnessy et al, 2000).

### Efficacy in metastatic breast cancer patients

Capecitabine has been investigated in an open-label, multicentre, phase II trial of patients (163 patients enrolled, 162 patients treated) with locally advanced or metastatic breast cancer whose disease had progressed with previous paclitaxel chemotherapy (Blum et al, 1999). Patients included in the trial were heavily pretreated: 91% had received prior anthracycline treatment, 82% had received prior 5-FU-based therapy and the mean number of prior chemotherapeutic agents was 4.7. Capecitabine (1255 mg/m$^2$) was administered twice daily for 14 days followed by a 7-day rest period. Patients who responded or had stable disease after 6 weeks' therapy (2 cycles) could continue treatment until disease progression or unacceptable toxicity.

The objective response rate for the study was 20% (32/162), which included 3 complete responses (Table 1). Stable disease was reported in 43% (70/162) of patients. Most responses occurred after the first 4 cycles of treatment. The response rate was consistent across a number of subpopulations, including those patients who received capecitabine as third-or fourth-line treatment (18% and 20%, respectively), showing that the drug is effective in a range of settings. Furthermore, in a subgroup of 42 patients with metastatic breast cancer that was both paclitaxel- and anthracycline-resistant, the objective response rate was 29%, which is particularly encouraging as response rates to salvage agents are usually lower in patients with established resistance.

The median duration of response was 7.9 months and median time to progression was 3.0 months in patients with measurable disease ($n$ = 135). Median survival time was 12.6 months for the entire population. This compares favourably with the 10.5-month median survival time reported by Valero et al (1998) for docetaxel in patients with paclitaxel-resistant disease.

In the capecitabine study, pain, analgesic consumption and performance status were documented using a 'Clinical Benefit Response' (CBR) score. Positive responses for the 3 individual parameters were defined as follows: ≥50% reduction in pain intensity in patients with baseline pain >20 mm on a visual analogue scale; ≥70 mg morphine equivalents per week; improvement of at least 20 points in Karnofsky Performance Status. A positive overall CBR was defined as a major improvement lasting for at least 4 consecutive weeks in at least one of the predefined

**Table 1** Efficacy in 3 phase II trials evaluating capecitabine as third-, second- and first-line treatment for metastatic breast cancer

| | Efficacy parameter | | |
|---|---|---|---|
| **Trial** | **Response rate (%)** | **Complete response rate (%)** | **Median TTP (months)** |
| Previous paclitaxel (Blum et al, 1999) | | | |
| Capecitabine ($n$ = 162) | 20 | 2 | 3.0 |
| Previous anthracyclines (RCT) (O'Reilly et al, 1998) | | | |
| Capecitabine ($n$ = 22) | 36 (95% Cl: 17–59%) | 14 | 3.0 |
| Paclitaxel ($n$ = 19) | 26 (95% Cl: 9–51%) | 0 | 3.1 |
| First-line (RCT) (Aapro, 2000) | | | |
| Capecitabine ($n$ = 61) | 30 (95% Cl: 19–43%) | 5 | 4.1 |
| CMF ($n$ = 32) | 16 (95% Cl: 5–33%) | 0 | 3.0 |

RCT = randomized, controlled trial; CMF = cyclophosphamide, methotrexate and 5-FU; TTP = time to disease progression.

*© 2001 Cancer Research Campaign*

parameters with all other parameters remaining stable. Overall CBR scores were positive (improvement in one parameter) for 29 of the 147 evaluable patients (20%) and 45 patients (31%) remained stable, regardless of ability to achieve a response. Cancer-related pain was reduced in 24 of the 51 patients (47%) who were able to achieve a positive response, i.e. >20 mm baseline pain, and opioid requirements were reduced in 22 of the 74 patients (30%) who were able to achieve a positive response.

Capecitabine was well tolerated in this study. Most adverse events were mild to moderate in intensity. Grade 3/4 toxicities included diarrhoea (14% of patients), hand-foot syndrome (10%, grade 3 only), fatigue (7%), nausea (4%), vomiting (4%) and stomatitis (3%). The incidence of neutropenia (3%) and neurotoxicity (1%) was low. No alopecia was reported, and hair growth occurred in some patients with alopecia at baseline. Grade 4 toxicity was reported in only 4% of patients. All adverse events were manageable by treatment interruption and, if necessary, dose reduction. Dose reduction did not affect efficacy. Adverse events led to withdrawal of 7% of women from the study, and there were no treatment-related deaths. These results demonstrate that capecitabine has high activity in heavily pretreated metastatic breast cancer and is well tolerated compared with other regimens.

The efficacy of capecitabine has also been investigated in an open-label, randomized, phase II study of patients with anthracycline-pretreated advanced breast cancer (O'Reilly et al, 1998). Paclitaxel was used as a reference arm in this study to ensure the absence of recruitment bias. Patients were randomized to receive one of 3 treatments: oral capecitabine either in a continuous schedule at a dose of 667 mg/m$^2$ twice daily on days 1–21 ($n$ = 2), or capecitabine in an intermittent schedule 1255 mg/m$^2$ twice daily on days 1–14 ($n$ = 22), or paclitaxel 175 mg/m$^2$ on day 1 ($n$ = 20) of a 3-week cycle.

Unfortunately, this study was discontinued prematurely because patients refused to be randomized since they had a clear preference for either an established intravenous cytotoxic or an investigational oral agent. The continuous regimen was discontinued following the selection of the intermittent regimen for further clinical development. However, the results obtained for capecitabine were encouraging (Table I). There were 3 complete responses and an overall objective response rate of 36% (95% CI: 17–59%) in patients receiving the intermittent schedule of capecitabine. An objective response rate of 26% (95% CI: 9–51%) was observed in patients receving paclitaxel, with no complete responses. Median time to progression was 3.0 months with capecitabine and 3.1 months with paclitaxel.

Grade 3/4 toxicity for treatment-related clinical adverse events was reported in 58% of patients receiving paclitaxel and in 23% receiving capecitabine. Likewise, the incidence of grade 3/4 neutropenia was higher in the paclitaxel group (53%) than in the capecitabine group (9%). Although this study was discontinued prematurely because of recruitment issues, the results suggest that capecitabine shows promising activity in patients with anthracycline-pretreated breast cancer.

Another randomized, phase II trial in metastatic breast cancer investigated the efficacy and tolerability of capecitabine as first-line treatment for metastatic breast cancer in women aged 55 years or older (Aapro, 2000). 95 patients were randomized (2:1) to either intermittent capecitabine treatment (1255 mg/m$^2$ twice daily for 14 days followed by a 7-day rest period) or low-dose CMF (cyclophosphamide 400 mg/m$^2$, methotrexate 40 mg/m$^2$, 5-FU 600 mg/m$^2$) administered intravenously on day 1 of each 21-day cycle.

A response rate of 30% (95% CI: 19–43%) was observed in 61 patients treated with capecitabine, compared with 16% (95% CI: 5–33%) in the CMF group ($n$ = 32) (Table 1). 3 patients receiving capecitabine achieved complete responses, whereas there were no complete responses with CMF. The median survival time was 21.6 months with capecitabine compared with 17.2 months in patients receiving CMF. As a randomized, phase II study, this trial was not powered to identify a statistically significant difference in survival between the 2 treatment arms.

The main differences in the tolerability profile of capecitabine compared with CMF were a higher incidence of grade 3 hand-foot syndrome (15% vs 0%, respectively) and more cases of grade 3 diarrhoea (8% vs 3%, respectively, with no grade 4 diarrhoea in either group). However, both of these adverse events were generally of mild to moderate nature and could be managed by treatment interruption or dose adjustment if appropriate. The incidence of grade 3 or 4 neutropenia was considerably higher in patients treated with CMF (41% compared with only 8% in the capecitabine group), and alopecia also occurred more frequently with CMF than capecitabine.

The encouraging antitumour activity and tolerability profile observed with capecitabine in these trials has significant implications for treatment, both in first-and second-line regimens, and also in patients failing anthracyclines and/or taxoids. Capecitabine therapy may also result in fewer interventions and hospitalizations.

Other oral fluoropyrimidines are under active study but capecitabine is the dominant agent of interest, partly because it is the first of the class to be approved by the FDA in the USA and appears to be the nearest to licensing in Europe. 2 other agents, UFT and S1, are in clinical development. Both of these agents contain DPD inhibitors (uracil in UFT and 5-chloro-2, 4-dihydropyrimidine [CDHP] in S1), which complete directly with 5-FU for the uracil binding site of DPD. The effect of competitive DPD inhibition is to increase the proportion of 5-FU available for anabolism to cytotoxic compounds and reduce the proportion catabolized to inactive compounds. The clinical development of another DPD inhibitor, eniluracil, which had been investigated in combination with oral 5-FU, has recently been abandoned.

### CONCLUSIONS

Despite the development of new anti cancer agents in the last few years, the expectations of a significant impact on the treatment of advanced breast cancer clearly have not been fully met. There is a need for new therapies that are of comparable efficacy but better tolerated than the taxoids. These new agents would provide alternatives to taxoids both in first- and second-line regimens and for patients with breast cancer that is unresponsive or refractory to taxoids.

Until recently, the treatment alternatives to taxoids were very limited. However, results from studies in advanced breast cancer of the new, oral, enzymatically activated fluoropyrimidine, capecitabine, indicate that this agent may be useful when anthracycline-based chemotherapy fails as well as in taxoid failures. Capecitabine may offer an effective, well-tolerated and more convenient alternative to taxoids and other intravenous cytotoxic agents. Clinical trials of capecitabine in breast cancer have demonstrated substantial activity with durable responses and meaningful clinical benefits.

© 2001 Cancer Research Campaign

In addition to its role in taxoid-resistant breast cancer, capecitabine may be useful as second-line therapy in anthracycline-resistant metastatic breast cancer or as first-line treatment as an alternative to intravenous chemotherapy. The improvement of patients' quality of life achieved by using oral agents with similar efficacy but enhanced tolerability is a vital component of the care of cancer patients where the goal of treatment is palliation. Furthermore, the low incidence of myelosuppression makes capecitabine an attractive agent for use in the adjuvant setting and also for incorporation into combination regimens, either with conventional agents, for example epirubicin/doxorubicin, or agents such as the taxoids and vinorelbine.

Most recently, data from a large, randomized, phase III trial have shown that the addition of capecitabine to docetaxel in anthracycline-pretreated patients results in significantly superior overall survival compared with docetaxel monotherapy as well as significantly superior response rates and time to disease progression (O'Shaughnessy et al, 2000). Clearly capecitabine as monotherapy or in combination with other cytotoxic agents provides an important new treatment option for metastatic breast cancer, combining convenience and a favourable side-effect profile with high activity. New data from ongoing monotherapy and combination trials are eagerly awaited.

### REFERENCES

Aapro M (2000) First-line capecitabine treatment for metastatic breast cancer (MBC): updated data from a randomised phase II trial. *Proc ICACT*: S4–10

Archer CD, Lowdell C, Sinnett HD, English J, Khan S and Coombes RC (1998) Docetaxel: response in patients who have received at least two prior chemotherapy regimens for metastatic breast cancer. *Eur J Cancer* **34**: 816–819

Bishop JF, Dewar J, Toner GC, Tattersall MHN, Oliver IN, Ackland S, Kennedy I, Goldstein D, Gurney H, Walpole E, Levi J and Stephenson J (1997) Paclitaxel as first-line treatment for metastatic breast cancer. The Taxol Investigational Trials Group, Australia and New Zealand. *Oncology (Huntingt)* **11** (4 Suppl 3): 19–23

Blum JL, Jones SE, Buzdar AU, LoRusso PM, Kuter I, Vogel C, Osterwalder B, Burger HU, Brown CS and Griffin T (1999) A multicenter phase II study of capecitabine in paclitaxel-refractory metastatic breast cancer. *J Clin Oncol* **17**: 485–493

Budman DR, Meropol NJ, Reigner B, Creavan PJ, Lichtman SM, Berghorn E, Behr J, Gordon R, Osterwalder B and Griffin T (1998) Preliminary studies of a novel oral fluoropyrimidine carbamate: capecitabine. *J Clin Oncol* **16**: 1795–1802

Carlson RW (1998) Quality of life issues in the treatment of metastatic breast cancer. *Oncology (Huntingt)* **12** (3 Suppl 4): 27–31

Cassidy J, Dirix L, Bissett D, Reigner B, Griffin T, Allman D, Osterwalder B and Van Oosterom AT (1998) A phase I study of capecitabine in combination with oral leucovorin in patients with intractable solid tumors. *Clin Cancer Res* **4**: 2755–2761

Chan S, Friedrichs K, Noel D, Pinter T, Van Belle S, Vorobiof D, Durate R, Gil Gil M, Bodrogi I, Murray E, Yelle L, von Minckwitz G, Korec S, Simmonds P, Buzzi F, Gonzalez Mancha R, Richardson G, Walpole E, Ronzoni M, Murawsky M, Alakl M and Riva A for the 303 Study Group (1999) Prospective randomized trial of docetaxel versus doxorubicin in patients with metastatic breast cancer. *J Clin Oncol* **17**: 2341–2354

D'Andrea GM and Seidman AD (1997) Docetaxel and paclitaxel in breast cancer therapy: present status and future prospects. *Semin Oncol* **24**(Suppl 13): S13–27–S13–44

Degardin M, Bonneterre J, Hecquet B, Pion JM, Adenis A, Horner D and Demaille A (1994) Vinorelbine (navelbine) as a salvage treatment for advanced breast cancer. *Ann Oncol* **5**: 423–426

DeMario MD and Ratain MJ (1998) Oral chemotherapy: rationale and future directions. *J Clin Oncol* **16**: 2557–2567

Fumoleau P, Delgado FM, Delozier T, Monnier A, Gil Delgado MA, Kerbrat P, Garcia-Giralt E, Keiling R, Namer M, Closon MT, Goudier MJ, Chollet P, Lecourt L and Montcuquet P (1993) Phase II trial of weekly intravenous vinorelbine in first-line advanced breast cancer chemotherapy. *J Clin Oncol* **11**: 1245–1252

Hansen R, Quebbeman E, Beatty P, Ritch P, Anderson T, Jenkins D, Frick J and Ausman R (1987) Continuous 5-fluorouracil infusion in refractory carcinoma of the breast. *Breast Cancer Res Treat* **10**: 145–149

Hortobagyi GN and Piccart-Gebhart MJ (1996) Current management of advanced breast cancer. *Semin Oncol* **23**(Suppl 11): 1–5

Huan S, Pazdur R, Singhakowinta A, Samal B and Vaitevicius VK (1989) Low-dose continuous infusion 5-fluorouracil. Evaluation in advanced breast cancer. *Cancer* **63**: 419–422

Ishikawa T, Utoh M, Sawada N, Nishida M, Fukase Y, Sekiguchi F and Ishitsuka H (1998a) Tumour selective delivery of 5-fluorouracil by capecitabine, a new oral fluoropyrimidine carbamate, in human cancer xenografts. *Biochem Pharmacol* **55**: 1091–1097

Ishikawa T, Sekiguchi F, Fukase Y, Sawada N and Ishitsuka H (1998b) Positive correlation between the efficacy of capecitabine and doxifluridine and the ratio of thymidine phosphorylase to dihydropyrimidine dehydrogenase activities in tumours in human cancer xenografts. *Cancer Res* **58**: 685–690

Jabboury K, Holmes FA and Hortobagyi G (1989) 5-fluorouracil rechallenge by protracted infusion in refractory breast cancer. *Cancer* **64**: 793–797

Johnson MR, Hageboutros A, Wang K, High L, Smith JB and Diasio RB (1999) Life-threatening toxicity in a dihydropyrimidine dehydrogenase deficient patient after treatment with topical 5-fluorouracil. *Clin Cancer Res* **5**: 2006–2011

Jones S, Winer E, Vogel C, Laufman L, Hutchins L, O'Rourke M, Lembersky B, Budman D, Bigley J and Hoeneker J (1995) Randomized comparison of vinorelbine and melphalan in anthracycline-refractory advanced breast cancer. *J Clin Oncol* **13**: 2567–2574

Judson IR, Beale PJ, Trigo JM, Aherne W, Crompton T, Jones D, Bush E and Reigner B (1999) A human capecitabine excretion balance and pharmacokinetic study after administration of a single oral dose of $^{14}$C-labelled drug. *Invest New Drugs* **17**: 453–460

Khoury P, Villalona-Calero M, Blum J, Diab S, Elledge R, Kraynak M, Moczygemba J, Kromelis P, Morales I, Brown C, Griffin T, Von Hoff D and Rowinsky E (1998) Phase I study of capecitabine in combination with paclitaxel in patients with previously treated metastatic breast cancer. *Proc Am Soc Clin Oncol* **17**: 206a (Abstract 793)

Liu G, Franssen E, Fitch M and Warner E (1997) Patient preferences for oral versus intravenous palliative chemotherapy. *J Clin Oncol* **15**: 110–115

Livingston RB, Ellis GK, Gralow JR, Williams MA, White R, McGuirt C, Adamkiewicz BB and Long CA (1997) Dose-intensive vinorelbine with concurrent granulocyte colony-stimulating factor support in paclitaxel-refractory metastatic breast cancer. *J Clin Oncol* **15**: 1395–1400

Lu Z, Zhang R, Carpenter JT and Diasio RB (1998) Decreased dihydropyrimidine dehydrogenase activity in a population of patients with breast cancer: implication for 5-fluorouracil-based chemotherapy. *Clin Cancer Res* **4**: 325–329

Mackean M, Planting A, Twelves C, Schellens J, Allman D, Osterwalder B, Reginer B, Griffin T, Kaye S and Verweij J (1998) Phase I and pharmacologic study of intermittent twice daily oral therapy with capecitabine in patients with advanced and/or metastatic cancer. *J Clin Oncol* **16**: 2977–2985

Miwa M, Nishida UM, Ishikawa ST, Mori K, Umeda SI and Ishitsuka H (1998) Design of a novel oral fluoropyrimidine carbamate, capecitabine, which generates 5-fluorouracil selectively in tumours by enzymes concentrated in human liver and cancer tissue. *Eur J Cancer* **34**: 1274–1281

Nabholtz JM, Senn HJ, Bezwoda WR, Melnychuk D, Deschenes L, Douma J, Vandenberg TA, Rapoport B, Rosso R, Trillet-Lenoir V, Drbal J, Molino A, Nortier JW, Richel DJ, Nagykalnai T, Siedlecki P, Wilking N, Genot JY, Hupperets PS, Pannuti F, Skarlos D, Tomiak EM, Murawsky M, Alakl M and Aapro M for the 304 Study Group (1999) Prospective randomized trial of docetaxel versus mitomycin plus vinblastine in patients with metastatic breast cancer progressing despite previous anthracycline-containing chemotherapy. *J Clin Oncol* **17**: 1413–1424

Ng JS, Cameron DA and Leonard RCF (1994) Infusional 5-fluorouracil in breast cancer. *Cancer Treat Rev* **20**: 357–364

O'Reilly SM, Moiseyenko V, Talbot DC, Gordan RJ, Griffin T and Osterwalder B (1998) A randomized phase II study of Xeloda™ (capecitabine) vs paclitaxel in breast cancer patients failing previous anthracycline therapy. *Proc Am Soc Clin Oncol* **17**: 163a (Abstract 627)

O'Shaughnessy J, Vukelja S, Moiseyenko V, Miles D, Cervantes G, Chan NavarroC, Mauriac L, Van Hazel G, Lui W-Y, Ayoub J-P, Rizel S, Osterwalder B, Rutman O and Leonard R (2000) Results of a large phase III trial of Xeloda®/Taxotere® combination therapy vs Taxotere® monotherapy in metastatic breast cancer

© 2001 Cancer Research Campaign

(MBC) patients. Poster presented at the San Antonio Breast Cancer Symposium, San Antonio, USA; (Abstract 381).

Pronk L, Vasey AP, Sparreboom A, Reigner B, Planting ASTh, Gordon RJ, Osterwalder B, Verweij J and Twelves C (2000) A phase I and pharmacokinetic study of the combination of capecitabine and docetaxel in patients with advanced solid tumours. *Br J Cancer* **83**: 22–29

Ragaz J, Campbell C, Gelmon K, Tolcher T, Bryce C and O'Reilly S (1997) Can patients with metastatic breast cancer (MBC) pretreated with cyclophosphamide (C), anthracyclines (A) or Taxol (Tax) respond to infusions of 5-FU (FUI)? Rationale for FUI-containing chemotherapy (CT) regimens. *Breast Cancer Res Treat* **46**: 95 (Abstract 411)

Ray-Coquard I, Biron P, Bachelot T, Guastalla JP, Catimel G, Merrouche Y, Droz JP, Chauvin F and Blay JY (1998) Vinorelbine and cisplatin (CIVIC regimen) for the treatment of metastatic breast carcinoma after failure of anthracycline- and/or paclitaxel-containing regimens. *Cancer* **82**: 134–140

Roché H, Fumoleau P, Tresca P, Pinon G, Serin D, Marie FN, Delgado M and Belpomme D (1990) Vinorelbine, a new active drug in breast carcinoma: results of an ARTAC phase II trial. *Ann Oncol* **1**(Suppl): 36

Romero A, Rabinovich MG, Vallejo CT, Perez JE, Rodriguez R, Cuevas MA, Machiavelli M, Lacava JA, Langhi M, Romero Acuna L, Amato S, Barbieri R, Sabatini C and Leone BA (1994) Vinorelbine as first-line chemotherapy for metastatic breast carcinoma. *J Clin Oncol* **12**: 336–341

Schiff PB and Horwitz SB (1980) Taxol stabilizes microtubules in mouse fibroblast cells. *Proc Natl Acad Sci USA* **77**: 1561–1565

Schüller J, Cassidy J, Dumont E, Roos B, Durston S, Banken L, Utoh M, Mori K, Weidekamm E and Reigner B (2000) Preferential activation of capecitabine in tumor following oral administration in colorectal cancer patients. *Cancer Chemother Pharmacol* **45**: 291–297

Seidman AD, Tiersten A, Hudis C, Gollub M, Barrett S, Yao T-J, Lepore J, Gilewski T, Currie V, Crown J, Hakes T, Baselga J, Sklarin N, Moynihan ME, Tong W, Egorin M, Kearns C, Spriggs D and Norton L (1995) Phase II trial of paclitaxel by 3-hour infusion as initial and salvage chemotherapy for metastatic breast cancer. *J Clin Oncol* **13**: 2575–2581

Valero V, Jones SE, Von Hoff DD, Booser DJ, Mennel RG, Ravdin PM, Holmes FA, Rahman Z, Schottstaedt MW, Erban JK, Esparza-Guerra L, Earhart RH, Hortobagyi GN and Burris HA 3rd (1998) A phase II study of docetaxel in patients with paclitaxel-resistant metastatic breast cancer. *J Clin Oncol* **16**: 3362–3368

Van Cutsem E, Findlay M, Osterwalder B, Kocha W, Dalley D, Pazdur R, Cassidy J, Dirix L, Twelves C, Allman D, Burger HU and Verweij J (2000) Capecitabine (Xeloda™), an oral fluoropyrimidine carbamate with substantial activity in advanced colorectal cancer: results of a randomized phase II study. *J Clin Oncol* **18**: 1337–1345

Villalona-Calero MA, Weiss GR, Burris HA, Kraynak M, Rodrigues G, DrenglerRL, Eckhardt SG, Reigner B, Moczygemba J, Burger HU, Griffin T, Von Hoff DD and Rowinsky EK (1999) Phase I and pharmacokinetic study of the oral fluoropyrimidine capecitabine in combination with paclitaxel in patients with advanced solid malignancies. *J Clin Oncol* **17**: 1915–1925

Weber BL, Vogel C, Jones S, Harvey H, Hutchins L, Bigley J and Hohneker J (1995) Intravenous vinorelbine as first-line and second-line therapy in advanced breast cancer. *J Clin Oncol* **13**: 2722–2730

 *© 2001 Cancer Research Campaign*
